# Validation of Non-invasive Language Mapping Modalities for Eloquent Tumor Resection: A Pilot Study

**DOI:** 10.3389/fnins.2022.833073

**Published:** 2022-03-01

**Authors:** Matthew Muir, Rajan Patel, Jeffrey Traylor, Dhiego Chaves de Almeida Bastos, Sarah Prinsloo, Ho-Ling Liu, Kyle Noll, Jeffrey Wefel, Sudhakar Tummala, Vinodh Kumar, Sujit Prabhu

**Affiliations:** ^1^Department of Neurosurgery, The University of Texas MD Anderson Cancer Center, Houston, TX, United States; ^2^Department of Neurosurgery, Baylor College of Medicine, Houston, TX, United States; ^3^Department of Neurological Surgery, The University of Texas Southwestern Medical Center, Dallas, TX, United States; ^4^Department of Neurosurgery, Cleveland Clinic, Cleveland, OH, United States; ^5^Department of Imaging Physics, The University of Texas MD Anderson Cancer Center, Houston, TX, United States; ^6^Department of Neuropsychology, The University of Texas MD Anderson Cancer Center, Houston, TX, United States; ^7^Department of Neuro-Oncology, The University of Texas MD Anderson Cancer Center, Houston, TX, United States; ^8^Department of Neuroradiology, The University of Texas MD Anderson Cancer Center, Houston, TX, United States

**Keywords:** functional MRI, transcranial magnetic stimulation, direct cortical stimulation, gliomas, brain mapping, eloquent, aphasia

## Abstract

Many studies have established a link between extent of resection and survival in patients with gliomas. Surgeons must optimize the oncofunctional balance by maximizing the extent of resection and minimizing postoperative neurological morbidity. Preoperative functional imaging modalities are important tools for optimizing the oncofunctional balance. Transcranial magnetic stimulation (TMS) and functional magnetic resonance imaging (fMRI) are non-invasive imaging modalities that can be used for preoperative functional language mapping. Scarce data exist evaluating the accuracy of these preoperative modalities for language mapping compared with gold standard intraoperative data in the same cohort. This study compares the accuracy of fMRI and TMS for language mapping compared with intraoperative direct cortical stimulation (DCS). We also identified significant predictors of preoperative functional imaging accuracy, as well as significant predictors of functional outcomes. Evidence from this study could inform clinical judgment as well as provide neuroscientific insight. We used geometric distances to determine copositivity between preoperative data and intraoperative data. Twenty-eight patients were included who underwent both preoperative fMRI and TMS procedures, as well as an awake craniotomy and intraoperative language mapping. We found that TMS shows significantly superior correlation to intraoperative DCS compared with fMRI. TMS also showed significantly higher sensitivity and negative predictive value than specificity and positive predictive value. Poor cognitive baseline was associated with decreased TMS accuracy as well as increased risk for worsened aphasia postoperatively. TMS has emerged as a promising preoperative language mapping tool. Future work should be done to identify the proper role of each imaging modality in a comprehensive, multimodal approach to optimize the oncofunctional balance.

## Introduction

Several studies have established a survival advantage to gross total resection in patients with intracranial malignancies (Molinaro et al., 2020). However, lesions in perieloquent language locations represent a unique challenge given risk of surgically acquired cognitive–linguistic deficits (Berger et al., 1994). The surgeon must balance an aggressive resection with preservation of eloquent structures in order to maximize survival as well as postoperative long term language function. Accumulating data has established that anatomy alone cannot predict functionality, indicating the need for functional mapping (Pouratian and Bookheimer, 2010). The introduction of direct cortical stimulation (DCS) during awake craniotomies has revolutionized this effort, allowing the surgeon to interrogate cortical language function by introducing transient electrical lesions (Ojemann et al., 2008). However, functional data can only be obtained intraoperatively, prohibiting the use of DCS for patient selection or surgical planning (Ojemann et al., 2008). In addition, various factors including young age, mental illness, and conscious sedation intolerance exclude patients for consideration for an awake craniotomy (Picht et al., 2013; Tarapore et al., 2013). Furthermore, this approach is invasive and associated with a risk of intraoperative seizures that can lead to an aborted operation (Serletis and Bernstein, 2007; Nossek et al., 2013a,b; Gonen et al., 2014; Eseonu et al., 2018). These considerations indicate the need for accurate preoperative mapping modalities.

Transcranial magnetic stimulation (TMS) has emerged as a method of non-invasively applying inhibitory stimulation to the cortex. The addition of stereotactic navigation to TMS has introduced the possibility of using TMS for preoperative lesion based cortical mapping (Ille et al., 2015a). Studies have shown an excellent correlation of TMS to DCS for motor mapping (Vitikainen et al., 2009; Picht et al., 2011; Krieg et al., 2012; Tarapore et al., 2012). In addition, studies have demonstrated the accuracy of preoperative imaging modalities such as electroencephalography and magnetoencephalography (Tarapore et al., 2012; Babajani-Feremi et al., 2014). However, the data analyzing TMS correlation with DCS for language mapping are sparse and limited to small patient cohorts (Picht et al., 2013; Tarapore et al., 2013). Functional magnetic resonance imaging (fMRI) is a preoperative mapping modality traditionally used for preoperative functional mapping (Picht et al., 2013). Because of a variety of proposed mechanisms, recent studies have shown poor correlation between fMRI and the gold standard of DCS for language mapping, especially in glioma patients (Giussani et al., 2010).

Data exploring the accuracy of both TMS and fMRI for language mapping in the same patient cohort would provide valuable insight into the proper role of each in a comprehensive multimodal mapping approach. Here we report our experience with preoperative language mapping using TMS compared with fMRI and intraoperative DCS in a pilot study of 28 patients.

## Materials and Methods

### Inclusion and Exclusion Criteria

This study was approved by the institutional review board at our institution. Twenty-eight patients were included in this retrospective study with the following inclusion criteria: presence of left-sided glioma close to language eloquent structures according to Sawaya et al. (1998), underwent preoperative language mapping and awake craniotomy with DCS language mapping, and at least 18 years of age. Exclusion criteria consisted of severe aphasia per clinical neurological examination or contraindications to TMS mapping such as a pacemaker or cochlear implant (Rossi et al., 2009). All patients underwent preoperative TMS language mapping using an object-naming task paradigm up to a week before surgery and preoperative fMRI language mapping using three paradigms: sentence completion, letter fluency, and category fluency tasks. They also underwent preoperative neuropsychological evaluation.

### Language Assessment

Preoperative language functioning was assessed within 1 week of planned surgical intervention with four different neuropsychological tests: Boston Naming Test (BNT), semantic fluency (Animals), Multilingual Aphasia Examination Controlled Oral Word Association (COWA), and the Multilingual Aphasia Examination Token Test (Token) (Spreen and Strauss, 1998). The BNT is an object naming task assessing expressive language, Animals and COWA are also expressive language measures interrogating lexical and semantic verbal fluency respectively, and Token is a measure of auditory comprehension aspects of receptive language. Patient performances on each task were converted to *z* scores (mean = 0, SD = 1) based on normative data stratified by age and education [see Noll et al. (2015) for sources of normative data]. Per common convention in neuropsychological research, a *z* score ≤−1.5 was considered impaired. Frank postoperative aphasia was diagnosed *via* clinical neurologic examination and gathered from the postoperative clinical notes by the neurosurgery team in the week after surgery and again at 30-day follow-up. For appropriate comparison to postoperative status, binary measures of preoperative aphasia (i.e., present or absent) were also collected from the patient’s chart. Postoperative aphasia immediately after surgery and that at 30-day follow-up were all recorded relative to preoperative aphasia and qualitatively recorded as the following categorical variables: “worsened” and “unchanged or improved” per record review.

### Transcranial Magnetic Stimulation Mapping Protocol

A navigated TMS system was utilized in the present study (NBS System 3.2; Nexstim, Helsinki, Finland). TMS stimulation onset was simultaneous with presentation of the line drawing object (picture to trigger interval was 0.0 s). Time-locked rapid rate TMS trains were given for 5 Hz/5 pulses for 140 stimulations. The display time was 1,000 ms, and interpicture interval was 3,000 ms. We performed TMS language mapping according to the conventionally accepted protocol for TMS language mapping (Lioumis et al., 2012; Picht et al., 2013; Sollmann et al., 2013). The most likely location of the hand knob was identified anatomically. This area was then stimulated in a random pattern while systematically varying the rotation, tilt, and yaw of the magnetic field. The location of maximal motor evoked potential was identified. Resting motor threshold (RMT) was identified using this position (Krieg et al., 2012). Once determined, the individual’s RMT was used as a basic value for the repetitive TMS language mapping procedure (Lioumis et al., 2012; Krieg et al., 2013; Picht et al., 2013; Sollmann et al., 2013). The patient then was baselined without stimulation on the object naming task, which consisted of presentation of line drawings of common objects and required the patient to verbally respond with the name of the pictured object. We used a common anterior/posterior coil orientation where the coil is oriented horizontally between the nasion and acoustic meatus. Pictures that elicited misnaming or hesitation were discarded. Baselining was repeated until a consistent baseline was obtained. Target dots 1 cm apart from each other were created in the surface of the three-dimensional (3D) brain reconstruction, based on the preoperative MRI, in a grid-like pattern. One centimeter was selected because a 1-cm radius of tissue is preserved around intraoperatively identified language-positive sites. The grid was created in the tumoral and peritumoral area. The pictures used on baseline were presented time locked to the TMS pulses while the coil was moved over the cerebral hemisphere. Each target dot was stimulated between four and five times non-consecutively. During this process, the patient was audiovisual-recorded. The audiovisual recordings were analyzed offline by the examiner who was also blind to the location of each stimulus in the 3D reconstruction. A site was considered a positive “hit” for language eloquence if disruption (e.g., speech arrest, hesitation, and paraphasic error) was observed on at least two of the trials. A hesitation was counted when the patient’s response came after the TMS stimulation had ended for that picture. Positive sites were marked in the 3D brain surface as white dots, and this information was sent as a report to the surgeon. These sites were also exported in digital imaging and communications in medicine (DICOM) files to be uploaded in the Neuronavigation system (Elements; BrainLab, Munich, Germany).

### Functional Magnetic Resonance Imaging Protocol

Magnetic Resonance Imaging scans were acquired on 3-T clinical scanners (GE Healthcare, Milwaukee, WI, United States), including fMRI scans using a T2*-weighted gradient-echo echo-planar imaging sequence (repetition time/echo time = 2,000/25 ms; matrix size = 64 × 64; field of view = 24 cm × 24 cm; 32 slices with 4-mm thickness and no gap; in-plane resolution = 3.75 mm^2^ × 3.75 mm^2^), and high-resolution T_2_-weighted fluid-attenuated inversion recovery and 3D spoiled gradient-echo T1-weighted sequences were acquired for anatomic reference.

The fMRI scans included three task paradigms: letter fluency (LETT), category fluency (CAT), and sentence completion (SENT). All paradigms consisted of control and task blocks alternating for 20 s each. The task paradigms included six cycles of 20-s control and 20-s task blocks. For LETT, patients were presented with a letter of the alphabet and asked to generate words that started with that letter during each task block. For the control block, participants were asked to tap their index finger on their thumb bilaterally. For CAT, patients were presented with a category (e.g., cities or types of food) and asked to generate words related to the category. For the control block, participants were again asked to tap their index finger on their thumb bilaterally. For SENT, the task blocks consisted of incomplete sentences for which patients were asked to fill in a blank. For the control block, participants were given four gibberish sentences with a format resembling that in the active block (each lasting 5 s). Before the fMRI, all patients underwent practice trials according to our standard preoperative mapping guidelines to ensure that the patient could perform the tasks correctly. During fMRI acquisition, each paradigm was displayed using an MRI-compatible 32-inch liquid crystal display, and oral instructions were provided through an intercom.

The fMRI data were processed by using the DynaSuite Neuro software, version 3.0 (*Invivo*; Philips, Gainesville, FL, United States). Image preprocessing for fMRI included motion correction and spatial smoothing with a 4-mm full-width-at-half-maximum Gaussian kernel. A functional activation map was created using the correlation analysis of the task paradigms convolved with a canonical hemodynamic response function and the signal intensity time course for each voxel. Statistical thresholds ranging from corrected *p* < 10^–6^–10^–2^ were applied to optimize the visualization of language areas.

### Neuronavigation and Surgical Planning

**FIGURE 1:**
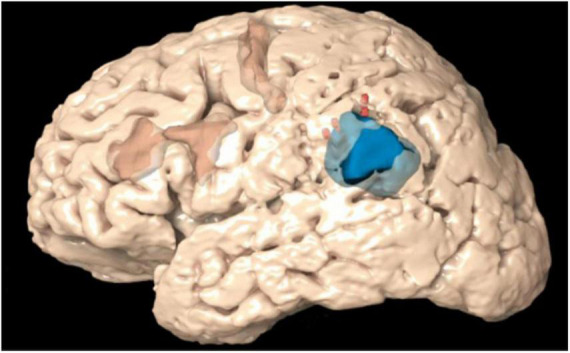
3D reconstruction of a preoperative plan depicting the transcranial magnetic stimulation (TMS) positive points (red) and blood oxygen level–dependent (BOLD) signal (orange) in context of the tumor (blue).

Before the surgery, TMS points were imported to BrainLab Elements in DICOM image format. Processed fMRI data with blood oxygen level–dependent (BOLD) signal were sent to BrainLab Elements from the electronic medical records (EPIC). 3D objects were made of the TMS points as well as fMRI BOLD signal. The functional data were then coregistered to the preoperative structural MRI to create a comprehensive preoperative plan and sent to the surgeon. Figure 1 shows an example of a 3D reconstruction of a preoperative plan.

### Direct Cortical Stimulation

Following craniotomy and opening the dura, the patient was awakened with the removal of the laryngeal mask and was able to communicate with the neuroanesthesiologist and the surgeon *via* a microphone. Prior to stimulation, when the patients were deemed sufficiently alert, they were asked to perform an object naming task using picture cards for baselining. All the tasks were performed by either a neuropsychologist (KN) or a speech pathologist (JP) to facilitate the interpretation of errors during stimulation. Once a consistent baseline was established, patients were then asked to perform the same task during DCS. DCS was conducted by the surgeon synchronized with the tester. A leading phrase (“This is a…”) would prompt the stimulation for approximately 2 s per stimulus starting at 1 mA. This process was repeated to stimulate the entire exposed surface of the brain. In addition, the patient was asked to generate words using phonemic and semantic categories similar to the fMRI verbal fluency paradigms. Any speech hesitation, dysnomia, paraphasic errors, and speech arrest were observed and noted. A site was considered a “hit” for language eloquence if any performance aberration was noted on at least two of three stimulations of the same site. An Ojemann stimulator (Radionics Inc., Burlington, MA, United States) was used with 5-mm spacing between the electrodes. For localization of the primary language and motor cortex, the stimulus was applied in increments of 1 mA, starting at 0.5 mA. The maximum stimulus needed to localize the language center was typically 4–6 mA. Positive DCS points were acquired using BrainLab and saved for offline analysis.

### Data Analysis

The positive and negative TMS points were imported into the navigation software (BrainLab Elements) for analysis. Separate 3D objects were made of both the positive and negative points. The DICOM image was fused with the intraoperative T1 MRI containing the positive DCS points as well as a postoperative MRI. A 3D reconstruction of the craniotomy was generated from the postoperative MRI. TMS points lying outside the resection cavity were excluded from analyses as these areas were not examined intraoperatively with DCS. We recorded the relevant coordinates (negative TMS points, positive TMS points, and DCS points) from the resulting plan for statistical analysis.

**FIGURE 2:**
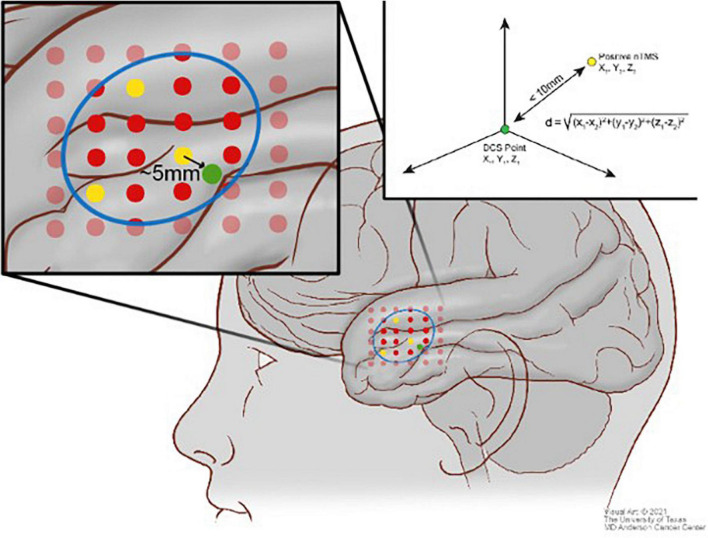
Scheme illustrating the comparison between the direct cortical stimulation (DCS) points (green) and the transcranial magnetic stimulation (TMS) positive points (yellow) and TMS negative points (red) using the distance equation in context of the craniotomy (blue).

Each positive TMS point was systematically measured for proximity to positive DCS points. A distance equation was utilized to determine if any DCS-positive points were located geometrically within 10 mm, which was considered a good correspondence between TMS and DCS. A distance of 10 mm was chosen because it is the widely accepted standard of error for DCS during language mapping (Haglund et al., 1994; Tharin and Golby, 2007; Sanai et al., 2008; Szelényi et al., 2010). Similar methodology was used for negative TMS points. If there were no positive DCS points within 10 mm, the point was counted as a true negative. If there was a positive DCS point within 10 mm of a negative TMS point, the point was counted as a false negative. In order to avoid labeling a portion of the cortex as both TMS positive and TMS negative, any point within 10 mm of a TMS-positive point was excluded from the analysis. Using DCS as the ground truth, positive predictive value (PPV), negative predictive value (NPV), sensitivity, and specificity were calculated from these values. Figure 2 illustrates the visual analytic process for TMS compared with DCS.

**FIGURE 3:**
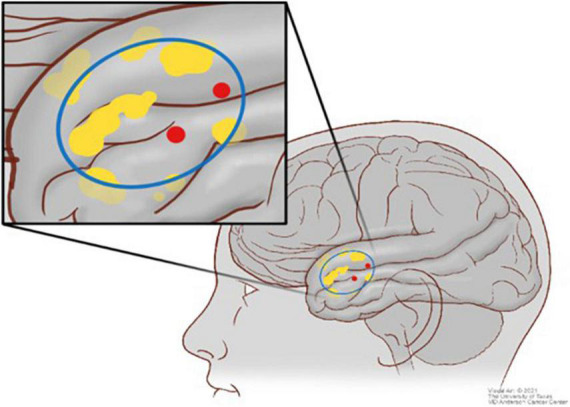
Scheme illustrating the comparison between the direct cortical stimulation (DCS) points (red) and the blood oxygen level–dependent (BOLD) signal (yellow) in context of the craniotomy (blue).

For fMRI analysis, 10-mm spheres were manually drawn around positive DCS points. DCS spheres were visually inspected for overlap with fMRI BOLD signal for any of the fMRI paradigms utilized. If there was no BOLD signal within the craniotomy and also no positive points, a true negative was recorded. If there was BOLD signal within the craniotomy overlapping with a DCS sphere, a true positive was recorded. If there was BOLD signal within the craniotomy but separate from a DCS positive sphere, both a false positive and a false negative were recorded. Again, having DCS as ground truth, PPV, NPV, sensitivity, and specificity were calculated from these values. Figure 3 illustrates the visual analytic process for fMRI compared with DCS.

### Statistical Analysis

Analyses were performed with SPSS 24 (IBM, Armonk, NY, United States). True positives, false positives, true negatives, and false negatives for the entire cohort were added. We used these data to analyze each modality as binary classifiers in relation to DCS prediction using a single threshold of 10 mm, the standard error for both TMS and fMRI. Contingency tables were constructed to model the predictive value of both fMRI and TMS in relation to intraoperative DCS data and are shown in Table 1. The receiver operating characteristics sensitivity, specificity, NPV, and PPV were calculated for both modalities. Binary logistic regression was performed for each modality, and statistical significance was evaluated using the Hosmer-Lemeshow goodness-of-fit test at a significant level of *p* < 0.05.

**TABLE 1:** Confusion matrices for transcranial magnetic stimulation (TMS) versus direct cortical stimulation (DCS) (left) and functional magnetic resonance imaging (fMRI) versus DCS (right).

	DCS^+^	DCS^–^		DCS^+^	DCS^–^
TMS^+^	27	146	fMRI+	5	12
TMS^–^	4	302	fMRI−	2	8
The sensitivity of TMS for language mapping was 87%, specificity was 67%, positive predictive value was 16%, and negative predictive value was 99%.			The sensitivity of fMRI for language mapping was 71%, specificity was 40%, positive predictive value was 29%, and negative predictive value was 80%.		

Sensitivity of the preoperative modality (TMS or fMRI) represents the ability of the preoperative modality to correctly identify language-positive sites as determined by the ground truth intraoperative DCS. Specificity refers to the ability to correctly identify language-negative sites. PPV refers to the probability that language-positive site in terms of preoperative modalities will be defined as language positive by DCS. NPV refers to the probability that language-negative sites in terms of preoperative modalities will be defined as language negative by DCS.

χ^2^ Tests were used to evaluate for preoperative factors that could be predictive of postoperative aphasia, such as age, location, cognitive status, or TMS variables. These variables were binarized. Age was binarized according to the median age (52). The total number of positive TMS points for each patient was also binarized according to the median (5). Location was binarized according to location (anterior vs. posterior). Tumor grade was binarized according to low- and high-grade tumors. Cognitive status was binarized according to impaired versus not impaired (*z* < −1.5 is impaired). Pearson product–moment correlation tests were used to evaluate preoperative factors such as tumor location (anterior vs. posterior), tumor grade, and baseline neuropsychological language functioning that could be associated with TMS performance. Tumors were divided into anterior and posterior groups to evaluate for location-dependent accuracy of TMS. Tumors anterior to the terminal end of the central sulcus were defined as anterior and tumors posterior to the terminal end were defined as posterior. Given the exploratory nature of the study, an unadjusted *p* ≤ 0.05 was considered statistically significant.

## Results

Table 2 shows the patient characteristics, including preoperative and postoperative measures of aphasia. Twenty-eight patients were enrolled with surgeries between January 2017 and December 2018. The ages ranged from 25 to 71 years (mean = 52 years, SD = 16 years). Five patients (18%) had a diffuse astrocytoma World Health Organization grade II, 10 patients (36%) had an anaplastic astrocytoma grade III, and 14 patients (50%) had a glioblastoma.

**TABLE 2:** Patient characteristics.

Patient	Age (y)	Gender	Tumor histology	Location	Handedness	Preoperative aphasia	Postoperative aphasia?	Postoperative aphasia compared with preoperative	Postoperative aphasia at 1 month	1 month compared with preoperative
1	57	Female	Glioblastoma	Left temporal	Right	Yes	Yes	Aggravated	Yes	Aggravated
2	46	Female	Anaplastic astrocytoma	Left frontal	Right	No	Yes	Aggravated	Yes	Aggravated
3	45	Male	Diffuse glioma	Left temporal	Right	Yes	Yes	Aggravated	Yes	Aggravated
4	57	Male	Glioblastoma	Left temporoparietal	Right	Yes	Yes	Aggravated	Yes	Aggravated
5	28	Male	Anaplastic astrocytoma	Left temporal	Mixed	No	Yes	Aggravated	Yes	Aggravated
6	63	Female	Anaplastic oligodendroglioma	Left frontal	Right	No	Yes	Aggravated	Yes	Aggravated
7	60	Male	Glioblastoma	Left frontal	Right	No	Yes	Aggravated	Yes	Aggravated
8	69	Female	Anaplastic astrocytoma	Left frontal	Right	No	Yes	Aggravated	Yes	Aggravated
9	63	Female	Glioblastoma	Left temporal	Right	Yes	Yes	Aggravated	Yes	Aggravated
10	34	Male	Anaplastic astrocytoma	Left temporal	Right	Yes	Yes	Unchanged	Yes	Unchanged
11	58	Male	Glioblastoma	Left temporal	Right	Yes	Yes	Unchanged	No follow-up	N/A
12	35	Male	Anaplastic astrocytoma	Left temporal	Right	No	No	Unchanged	No	Unchanged
13	66	Female	Anaplastic oligodendroglioma	Left insular/temporal	Mixed	Yes	Yes	Aggravated	Yes	Unchanged
14	60	Male	Anaplastic astrocytoma	Left frontal	Right	No	Yes	Aggravated	No	Unchanged
15	56	Male	Glioblastoma	Left temporal	Right	No	Yes	Aggravated	No	Unchanged
16	43	Male	Diffuse astrocytoma	Left temporal	Right	No	No	Unchanged	No	Unchanged
17	22	Female	Glioblastoma	Left temporal	Right	No	Yes	Aggravated	No	Unchanged
18	31	Male	Diffuse astrocytoma	Left frontal	Right	No	No	Unchanged	No	Unchanged
19	70	Male	Glioblastoma	Left temporal	Right	Yes	Yes	Unchanged	No follow-up	N/A
20	71	Male	Glioblastoma	Left temporal	Right	No	Yes	Aggravated	No	Unchanged
21	23	Female	Diffuse astrocytoma	Left frontal	Right	No	No	Unchanged	No	Unchanged
22	61	Male	Glioblastoma	Left temporal	Right	No	Yes	Aggravated	Yes	Unchanged
23	27	Female	Diffuse astrocytoma	Left parietal	Right	No	No	Unchanged	No	Unchanged
24	67	Female	Glioblastoma	Left frontal	Right	No	No	Unchanged	No	Unchanged
25	71	Female	Glioblastoma	Left frontal	Right	No	No	Unchanged	No	Unchanged
26	40	Male	Anaplastic oligodendroglioma	Left frontal	Right	No	Yes	Aggravated	No follow-up	N/A
27	58	Female	Glioblastoma	Left temporal	Right	No	Yes	Aggravated	No follow-up	N/A
28	64	Female	Glioblastoma	Left frontal	Right	No	No	Unchanged	No	Unchanged

Table 1 depicts contingency tables for both TMS and fMRI compared with DCS. The sensitivity of TMS was 83%, specificity was 67%, NPV was 99%, and PPV was 16%. The sensitivity of fMRI was 71%, specificity was 40%, NPV was 80%, and PPV was 29%. fMRI results were not used intraoperatively in four patients because of poor baseline limiting the patient’s ability to comply with language mapping tasks (not severe enough to be excluded from TMS or the study). Eleven patients had no BOLD within the bounds of the craniotomy, consistent with negative intraoperative mapping with no DCS-positive points.

Table 3 shows binary logistic regression performed with fMRI and TMS in relation to predicting intraoperative DCS points using a single threshold of 10 mm. TMS showed statistically significant prediction of DCS points [odds ratio (OR) = 15.1, *p* < 0.0001], whereas fMRI did not show statistically significant prediction (OR = 1.7, *p* = 0.59).

**TABLE 3:** Univariate statistical analysis performed for each preoperative modality in relation to predicting intraoperative direct cortical stimulation (DCS) points.

	Odds ratio	95% Confidence interval	*p*-value
TMS	15.1	5.2–43.8	<0.0001
fMRI	1.7	0.26–10.8	0.59

*TMS, transcranial magnetic stimulation; fMRI, functional magnetic resonance imaging.*

Eight patients (29%) had preoperative aphasia per clinical neurologic examination. However, 86% of patients had at least one impaired score out of the four neuropsychological language tests. Regarding individual measures, 54% of patients were impaired on each verbal fluency measure, and 43% were impaired on confrontation naming, whereas comprehension was relatively preserved, with only 14% falling in the impaired range. On average, performances were lowest for verbal fluency [Animals (mean = −1.69, SD = 1.23); COWA (mean = −1.63, SD = 0.93)] and naming [BNT (mean = −1.37, SD = 1.35)], whereas comprehension was generally within normal limits [Token (mean = −0.20, SD = 1.23)]. Seventeen patients (61%) had transient worsened postoperative aphasia compared with preoperative status based on chart review. Twenty-four patients had 30-day follow-up; nine (38%) of these had worsened postoperative aphasia lasting longer than 30 days. Postoperative neuropsychological performances were not available for the majority of patients and thus were not analyzed. TMS-positive points were resected in 13 patients (46%). Five of these patients (38%) had postoperative aphasia lasting longer than 30 days. Eight (29%) had transient postoperative aphasia that subsided by 30-day follow-up. χ^2^ Tests showed no association between resection of positive points and worsened postoperative aphasia, whether transient (*p* = 0.60) or persistent (> 30 days) (*p* = 0.96).

Table 4 summarizes preoperative variables along with TMS true positives, false positives, true negatives, and false negatives for each patient. A significant inverse correlation was found between baseline semantic fluency (animals) and false positives (*r*_26_ = −0.46, *p* = 0.013), indicating that patients with better baseline verbal fluency had fewer false-positive TMS results. Ten of 12 patients with impaired baseline semantic fluency (*z* < −1.5) had temporal tumors, while the other two had frontal lobe tumors. Eliminating patients with impaired baseline semantic fluency (*z* < −1.5) increased the specificity of TMS from 67 to 76% without significantly affecting the other measures.

**TABLE 4:** Preoperative variables used to perform univariate analysis of transcranial magnetic stimulation (TMS) performance along with false positives (FP), true positives (TP), true negatives (TN), and false negatives (FN) for each patient.

Patient	Age	Tumor grade	Tumor location	Boston naming test (*z* score)	Semantic fluency (*z* score)	Token test (*z* score)	MAE_COWA_Z (*z* score)	TP	FP	TN	FN
1	60	3	Posterior	−0.9	−1.6	0.4	−1.18	0	16	44	0
2	61	4	Anterior	−1.1	−0.8	−1.3	−2.13	0	10	18	0
3	57	4	Anterior	−3.3	−2	0.4	−3.12	0	4	46	0
4	63	4	Posterior	−1.4	−3.7	−0.7	−1.14	0	13	10	0
5	71	4	Anterior	−3	−3.7	0.9	−1.86	0	12	4	0
6	60	4	Anterior	−2.4	−1.4		−2.8	0	3	9	0
7	27	4	Anterior	−1.7	−2.7	0.9	−0.31	0	12	3	0
8	63	2	Anterior	−0.6	−3.2	−0.4	−2.22	7	6	0	0
9	56	3	Posterior	−3	−3.8	0.9	−1.93	0	8	19	0
10	22	4	Anterior	−3.3	−1.6	0.9	−0.73	1	3	8	0
11	46	4	Posterior	−3.3	−3.9	−1.3	−1.45	0	3	2	0
12	70	3	Posterior	−0.8	−0.9	−2	−2.5	3	5	4	2
13	31	4	Anterior	−0.7	−0.4	0.9	−0.86	0	3	15	0
14	23	2	Anterior	0.8	−0.2	0.9	−0.76	3	2	8	0
15	69	2	Posterior	−0.8	−2	−2.6	−3.53	0	4	7	0
16	35	3	Posterior	−2.9	−1.6	0.9	1.01	3	3	5	1
17	43	2	Anterior	−1.6	−1.1	−1.3	−1.72	0	3	0	0
18	58	4	Anterior	−0.7	0.1	−0.1	−1.38	0	0	11	0
19	57	4	Posterior	1.5	−1.8	−0.1	−0.82	1	4	13	0
20	66	3	Anterior	−2.5	−3.1	0.9	−1.72	0	4	3	0
21	45	2	Anterior	−0.2	−0.9	−1.3	−1.29	0	5	15	0
22	28	3	Posterior	N/A	−2.1	0.9	−1.4	5	5	3	0
23	58	4	Posterior	−1.7	0.2	−2.6	−1.4	1	2	11	1
24	64	4	Anterior	−0.6	−0.4	0.4	−1.52	0	3	15	0
25	67	4	Anterior	−1.4	−0.1	0.4	−1.66	1	6	12	0
26	40	3	Anterior	−2	−1.4	0.9	−1.72	2	3	1	0
27	71	4	Posterior	−0.9	−1.1	−2.6	−2.93	0	0	10	0
28	34	3	Anterior	1.5	−2.2	0.4	−2.54	0	4	6	0

No significant correlation was found between baseline naming on the BNT and number of true positives (*p* = 0.37), false positives (*p* = 0.62), or true negatives (*p* = 0.97). No significant correlation was found between baseline phonemic fluency (COWA) and false positives (*p* = 0.40), true positives (*p* = 0.38), or true negatives (*p* = 0.24). No significant correlation was found between baseline comprehension (Token) and false positives (*p* = 0.88), true positives (*p* = 0.12), or true negatives (*p* = 0.94). No significant correlation was found between age and false positives (*p* = 0.67), true positives (*p* = 0.53), or true negatives (*p* = 0.85). No significant correlation was found between tumor grade and false positives (*p* = 0.33), true positives (*p* = 0.074), or true negatives (*p* = 0.73). No significant correlation was found between location (anterior vs. posterior) and false positives (*p* = 0.22), true positives (*p* = 0.95), or true negatives (*p* = 0.74). False negatives were not included in the analysis because of their low occurrence.

The total number of positive TMS points was significantly correlated with persistent worsened postoperative aphasia (at 30-day follow-up) (*r*_26_ = −0.60, *p* = 0.001). However, the total number of positive DCS points was not significantly associated with persistent worsened postoperative aphasia (*p* = 0.62). A significant negative correlation was found between preoperative semantic fluency performance (animals) and risk for persistent worsened postoperative aphasia (*r*_26_ = −0.56, *p* = 0.003). No significant correlation was found between preoperative cognitive status assessed by the other three tests and risk for persistent postoperative aphasia.

## Discussion

Few studies have explored the correlation between preoperative TMS and intraoperative DCS, each with subtle differences in methodology. Tarapore et al. (2013) published a cohort of 12 patients with perisylvian glioma, finding sensitivity, specificity, and NPV greater than 90%. The authors spatially normalized each MRI and used a geometric distance of 10 mm to calculate copositivity between TMS and DCS sites. Picht et al. (2013) reported a sensitivity >80% and an NPV >90%, but low specificity and PPV. The authors used a cortical parcellation system to divide the cortex into functionally distinct regions and count TMS and DCS points as copositive if they fell within the same region. Jung et al. (2019) found TMS sensitivity greater than 60% and NPV greater than 70% using the same cortical parcellation system in a heterogeneous cohort of brain tumor patients.

A recent study by Bährend et al. (2020) found a sensitivity of 35%, specificity of 96%, an NPV of 96%, and a PPV of 16% using the geometric system. However, because of methodological differences, these results are difficult to interpret in the context of the previous studies. The authors noted a significantly increased number of stimulation sites compared with previous work (1,342 vs. 183 and 160), mainly due to larger craniotomies and smaller distances between stimulations. The dramatically increased number of stimulation sites led to a disproportionate amount of true negatives (1,138/1,342), which is the dominant factor when calculating specificity and NPV.

Results for TMS from our cohort showed an NPV exceeding 99% and a sensitivity of 83%, indicating that TMS consistently identifies language-positive sites defined by intraoperative mapping. However, the specificity and PPV were lower at 67 and 16%, respectively. These results support previous studies, providing further evidence for the utility of TMS for presurgical negative mapping. Sanai et al. (2008) were the first to report that intraoperative negative DCS mapping was sufficient to minimize long-term neurological deficits. Accumulating data showing the high sensitivity and NPV of TMS may point to a similar utility for preoperative negative TMS mapping. Ille et al. (2016) reported a case series of four patients who underwent resection for perieloquent lesions but were ineligible for an awake craniotomy. Only preoperative TMS was used for language mapping. At 3-month follow-up, none of the patients showed neurological sequelae, demonstrating the feasibility of resecting purely based on preoperative data (Ille et al., 2016).

Several possible reasons exist for the high sensitivity and high rate of false positives reported in most studies comparing TMS and DCS. Neuroimaging studies have demonstrated that TMS activates circuitry through transsynaptic spread, affecting downstream circuits outside the region of interest (Paus et al., 1997; Valero-Cabre and Pascual-Leone, 2005; Bestmann, 2008). Thus, a TMS-positive point may be the result of activation of a distant, functionally connected region, leading to false positives in non-essential locations. Preliminary studies using TMS for language lateralization would seem to support this hypothesis, as positive speech arrest sites were found in the non-dominant hemisphere (Pascual-Leone et al., 1991; Jennum et al., 1994; Michelucci et al., 1994; Epstein et al., 1999; Wassermann et al., 1999). Further study of optimal frequency, duration, and intensity of TMS may yield conditions that reduce this non-specific activation of non-essential intracortical pathways, resulting in fewer false positives (Picht et al., 2013). Other groups have explored the use of alternative language tests in order to reduce false positives, such as action naming and verb generation (Hernandez-Pavon et al., 2014; Hauck et al., 2015; Muthuraman et al., 2016).

Another reason for the high rate of false positives may be due to discrepancies between interpreting speech arrests during TMS or DCS stimulation. Intraoperative DCS speech arrests tend to be much more dramatic than TMS speech arrests, resulting in more ambiguity in calling TMS-positive points during stimulation. This could result in overcalling TMS-induced speech errors. Interestingly, better baseline semantic fluency, a measure of semantic network, and expressive language functioning, was associated fewer false positives on TMS. However, more than half the sample had baseline impairment in this domain. As such, increased false-positive rates for TMS may also be associated with baseline disruption of expressive language and semantic network integrity. Finally, it also possible that the higher rate of positive points observed in TMS could be due to identifying true language essential points that DCS did not identify. However, analyses of clinical sequelae of patients who underwent TMS-positive point resection do not support this idea. Tarapore et al. (2013) found that three of four of these patients had no permanent neurological deficit, whereas Picht et al. (2013) found that none of the 10 patients who had TMS-positive points resected showed permanent neurological deficits (Picht et al., 2013; Tarapore et al., 2013). Our cohort showed that 5 of the 13 patients with TMS-positive points resected showed worsened aphasia lasting longer than 30 days. However, there was no correlation between resection of positive points and neurological outcome.

Interestingly, patient outcomes with respect to resection of positive TMS points seem to vary in motor cases versus language cases. Insight into this would provide information regarding whether TMS-positive points are truly essential for function. An analysis of a cohort of 43 patients by Moser et al. (2017) showed a direct correlation between the resection of TMS-positive motor points in prefrontal brain areas and postoperative motor function, whereas no such correlation has been found in TMS language studies to date (Picht et al., 2013; Tarapore et al., 2013; Moser et al., 2017). This could be due to different underlying neurophysiological mechanisms responsible for language versus motor function. Studies have shown that if more than one positive language site has been identified by DCS, one of them can be removed without permanent neurological sequel (Duffau, 2006; Robles et al., 2008). Neurological mechanisms for language seem to be far more complex than that of motor. Information coordinating movement is passed unidirectionally through long fiber tracks originating in M1 and terminating in spinal a-motor neurons, with modulating input from various other regions (Ries et al., 2019). In contrast, the anatomic distribution of sender and recipient neurons for language is organized in a mosaic pattern of discrete points, facilitating bidirectional communication, and redundant pathways (Chang et al., 2015). This more complex organization of the language system may be responsible for the unclear necessity of positive TMS and DCS language sites when compared with positive motor sites.

We also evaluated preoperative variables that could be predictive of TMS performance as well as risk for persistent worsened postoperative aphasia, finding mixed results. We found no correlation between age or tumor grade and TMS accuracy, using the outcomes of false positives, true positives, and false negatives. As noted previously, preoperative cognitive status assessed by semantic fluency (animal test) was found to be significantly correlated with false positives. Presumably, the TMS mapping process would be more impacted by a poor baseline than DCS because of the more subtle nature of errors induced by TMS. A poor baseline could lead to overcalling errors during preoperative mapping and more false positives. Eliminating patients with impaired semantic fluency test increased the specificity of TMS to 76% without significantly affecting the other measures of sensitivity, PPV, or NPV.

The total number of TMS-positive points upon preoperative mapping was significantly associated with risk for worsened postoperative aphasia at 30-day follow-up. Interestingly, the total number of DCS-positive points was not significantly associated with risk for persistent worsened postoperative aphasia. In addition, preoperative cognitive status assessed by semantic fluency was significantly associated with risk for persistent worsened postoperative aphasia. It is possible that compromise in baseline semantic fluency is an indicator of lesion proximity to eloquent networks, thus elevating risk of postoperative decline. These findings show that poor baseline status, whether captured by neuropsychological testing or TMS, is associated with postoperative neurological decline. More data concerning the impact of preoperative variables on TMS performance as well as risk for postoperative deficits could aid patient selection and risk stratification. Because of its superior correlations compared with the other three tests, the utility of the semantic fluency test should be further explored in the context of presurgical TMS language mapping.

Data regarding the utility of fMRI for presurgical language mapping in glioma patients are conflicting. A review of nine language mapping studies comparing fMRI to DCS showed widely varying levels of correlation (Giussani et al., 2010). The sensitivity ranged from 59 to 100%, whereas the specificity ranged from 0 to 97%. However, the results are difficult to interpret considering the heterogeneous patient populations (gliomas vs. benign tumors) and widely varying methodologies (Giussani et al., 2010). Some hypothesize that studies showing discrepancies between fMRI and DCS reflect methodological differences, as well as the pathophysiology of intracerebral lesions (Giussani et al., 2010). Picht et al. found that analyses of raw fMRI data by separate examiners can lead to different results, probably because fMRI relies on statistical thresholds that can vary by the individual patient as well as institution (Paus et al., 1997). In addition, gliomas diffusely infiltrate the brain parenchyma, decreasing the contact rate between glia and capillary cells, in turn decreasing the oxygen extraction fraction. These changes reduce the BOLD signal, rendering fMRI less accurate in cases involving intracerebral lesions (Feindel and Perot, 1965; Ojemann et al., 1998; Ulmer et al., 2003; Ulmer et al., 2004).

Despite these concerns, fMRI has shown significant utility for presurgical workup in glioma patients. Several groups have found good correlation between fMRI and intraoperative DCS for localizing motor, somatosensory, and visual areas (Roux et al., 2003; Majos et al., 2005; Li et al., 2006). Roux et al. (2003) showed that all of the 22 intraoperatively identified positive language sites were correlated to preoperative fMRI. A recent meta-analysis showed that preoperative fMRI decreases postoperative neurological morbidity (Luna et al., 2021), whereas a recent review found that fMRI has shown excellent utility for lateralizing language dominance, superior to the Wada test for many patients (Qadri et al., 2021). It is possible that combining task-based fMRI with cerebrovascular reactivity mapping (CVR) may help overcome some of the issues related to vascular uncoupling in the presence of brain tumors, although the impact of CVR mapping upon the accuracy of fMRI compared with DCS has not been adequately investigated to date.

Because of the widely varying patient populations and methodologies used in fMRI preoperative language mapping studies, considerable interest has turned to direct comparisons between the two main available preoperative modalities of TMS and fMRI within the same patient cohort. DCS maps the cortex by producing a transient virtual lesion, whereas fMRI maps by detecting changes in regional blood flow and oxygen extraction (Giussani et al., 2010). fMRI activations often overlie sulci, which hinders the ability of fMRI to precisely delineate between areas critical for language function versus areas merely participating, obscuring which tissue must be preserved during surgery. This fundamental mechanistic difference between fMRI and the gold standard of DCS could account for much of the difficulty in correlating fMRI to DCS. TMS, like DCS, produces a transient virtual lesion and is seemingly unaffected by intracerebral lesions (Pascual-Leone et al., 1999; Sollmann et al., 2013). The mechanistic similarities of TMS to DCS could indicate that TMS has potential for superior correlation to intraoperative mapping data. While researchers have evolved fMRI protocols for many years, using TMS for presurgical mapping purposes is still in its infancy, except for a few specialized centers. More studies should further refine TMS language mapping protocols as well as optimize patient selection, with subsequent comparison to fMRI.

Ille et al. (2015b) were the first to show advantages of TMS for preoperative language mapping compared with fMRI within the same patient cohort. The authors emphasized the utility of fMRI for language studies in healthy patients but outlined the confounding impact of gliomas on fMRI data. However, our study seems to support these previous findings, showing higher sensitivity, specificity, and NPV in TMS compared with fMRI within the same patient cohort (Ille et al., 2015b). In addition, TMS showed statistically significant prediction of intraoperative DCS points, whereas fMRI did not. However, the statistical validity of these results remains in question because of the significant differences in data collection and aggregation between TMS and fMRI, leading to far more data points for TMS despite a similar number of patients. In addition, TMS lacks large, aggregated studies showing its clinical utility for preoperative language mapping (Luna et al., 2021). TMS also lacks comparable evidence to fMRI with respect to lateralizing language dominance (Qadri et al., 2021). While TMS has shown significant potential, the proper role of each modality for presurgical language mapping in brain tumor patients has yet to be determined.

Other groups have recently compared preoperative TMS language mapping to intraoperative DCS mapping. Our study has a few key methodological differences that should be noted. First, we utilized a geometric distance equation to detect and label copositive TMS and DCS locations instead of the cortical parcellation system used by Picht et al. (2013). Using the cortical parcellation system, the cortex is divided into functionally relevant divisions delineated by Corina et al. (2005). Two points are considered copositive only if they are in the same region, regardless of actual proximity. Although this approach may be beneficial for basic research, we believe that using geometric distance to determine copositivity is the more clinically relevant approach for a few reasons.

Intraoperative positive DCS points are treated as discrete points; the entire region is not avoided during resection. TMS points should be accordingly analyzed as discrete points in relation to DCS, instead of broader regions of interest, to achieve consistency. Furthermore, electrical stimulation studies have shown that language function resides in “mosaics” of connected points, often less than 1 cm, instead of broad clusters (Ojemann et al., 1989). Using the cortical parcellation system, detecting one of these points with TMS would inaccurately label the entire region as essential for language. Finally, the cortical parcellation system was derived from patients undergoing resection for epilepsy (Corina et al., 2005). The mass effect and cerebral edema produced by the tumor in patients undergoing glioma resection most likely distort the functionally defined anatomy derived from a different patient population.

Another methodological difference in our study was the exclusion of any TMS points outside of the craniotomy as well as negative TMS points within 10 mm of another positive TMS point. Preoperative TMS routinely stimulates the cortex in a wider distribution than the resection cavity exposes. Because the language-positive points identified by TMS were never tested by DCS, failure to exclude these points would falsely lower the specificity and PPV. Because we used a 10-mm margin of error between TMS and DCS points in the calculations, we eliminated negative TMS points within 10 mm of other positive TMS points. We wanted to avoid labeling a given point on the cortex as both language positive and language negative by TMS. It is unclear whether other groups using the geometric distance approach also performed this exclusion.

## Future Directions and Limitations

A significant methodological limitation in studies of this kind is the arbitrary selection of the threshold used to determine copositivity. Changing the threshold would change the calculated measures of accuracy. We chose 10 mm in accordance with Tarapore et al. (2012, 2013). Future studies should systematically vary the comparison threshold to document the effects on the accuracy of preoperative imaging modalities.

This study is limited by the retrospective nature of the data collection as well as a small sample size. A major limitation of studies focused on preoperative and intraoperative imaging modalities is the “brain shift” induced by the cerebral edema common to glioma patients. Once the skull flap has been removed, many patients experience a dramatic shift of the cortex in response to pressure differences and draining fluid (Gerard et al., 2017). This shift distorts the anatomy, producing a discrepancy between the preoperative MRI and intraoperative DCS point acquisition. This shift is even more pronounced when analyzing postoperative MRIs to investigate clinical sequelae of patients who had TMS-positive points resected. As a result, the veracity of the important analyses of these patients is questionable. Further work needs to be done to investigate innovative strategies to more accurately track the shift of the cortex from preoperative through postoperative imaging. In addition, further work stratifying the results based on anatomical regions as well as preoperative functional status could aid in optimal patient selection. Finally, further refining the TMS language mapping methodology could improve its clinical utility. Combining the TMS data with other modalities such as diffusion tensor imaging tractography could extend the map to subcortical structures. Future research should pursue methods to address the high rate of false positives, possibly by only reporting positive sites with multiple speech arrests at different stages of the procedure. Pursuing more objective methods of calling errors preoperatively during TMS stimulation could improve the correlation between modalities. Despite these limitations, our study provides valuable insight into the utility of presurgical mapping modalities, as well as novel methods of comparison.

## Conclusion

TMS showed superior correlation with DCS compared with fMRI for preoperative language mapping in glioma patients. TMS showed high NPV and sensitivity, but comparably lower specificity and PPV. A poor cognitive baseline is associated with decreased TMS accuracy, as well as increased risk for persistent worsening postoperative aphasia. Future studies minimizing the confounding factor of brain shift, optimizing patient selection, and normalizing methods of error calling during TMS stimulation could increase the specificity and clinical utility of TMS.

## Data Availability Statement

The original contributions presented in the study are included in the article/supplementary material, further inquiries can be directed to the corresponding author.

## Ethics Statement

The studies involving human participants were reviewed and approved by University of Texas MD Anderson Cancer Center Institutional Review Board (#2021-0856). The patients/participants provided their written informed consent to participate in this study.

## Author Contributions

MM and RP: conception and design, data analysis, writing, and editing. JT: data analysis and editing. DA and SPra: conception and design, data acquisition, and editing. SPri, VK, JW, and ST: data acquisition and editing. KN: conception and design, data analysis, and editing. All authors contributed to the article and approved the submitted version.

## Conflict of Interest

The authors declare that the research was conducted in the absence of any commercial or financial relationships that could be construed as a potential conflict of interest.

## Publisher’s Note

All claims expressed in this article are solely those of the authors and do not necessarily represent those of their affiliated organizations, or those of the publisher, the editors and the reviewers. Any product that may be evaluated in this article, or claim that may be made by its manufacturer, is not guaranteed or endorsed by the publisher.
